# The roles of impulsivity, comorbid ADHD, and borderline personality disorder in patients with bulimia nervosa

**DOI:** 10.1007/s40519-025-01713-8

**Published:** 2025-01-18

**Authors:** Susanne Gilsbach, Julia Leuchtenberger, Beate Herpertz-Dahlmann, Ulrich Voderholzer, Kerstin Konrad, Georg von Polier, Jochen Seitz, Manfred Fichter

**Affiliations:** 1 Department of Child and Adolescent Psychiatry, Psychosomatics and Psychotherapy, Medical Faculty, RWTH Aachen University, Aachen, Germany; 2 Schoen Clinic Roseneck, Prien, Germany; 3 Department of Psychiatry and Psychotherapy, LMU University Hospital, LMU Munich, Munich, Germany; 4 Child Neuropsychology Section, Department of Child and Adolescent Psychiatry, Psychosomatics and Psychotherapy, Medical Faculty, RWTH Aachen University, Aachen, Germany; 5 Department of Child and Adolescent Psychiatry, Psychotherapy and Psychosomatics, University Hospital, Leipzig, Germany; 6 Institute of Neuroscience and Medicine Brain and Behaviour, Forschungszentrum Jülich, Jülich, Germany; 7 Department of Child and Adolescent Psychiatry, Psychosomatics and Psychotherapy, University Hospital Essen, University of Duisburg-Essen, Essen, Germany; 8 Forschungszentrum Jülich Institut for Neuroscience and Medicine-Brain and Behavior (INM 7), Jülich, Germany

**Keywords:** Bulimia nervosa, ADHS, Borderline personality disorder, Comorbidity, RDoC, Impulsive behaviour

## Abstract

**Background:**

Bulimia nervosa (BN) is a serious mental illness with impulsivity as a cardinal symptom. Impulsivity contributes to various other, often comorbid, mental disorders, such as attention deficit/hyperactivity disorder (ADHD) and borderline personality disorder (BPD). The aim of this study was to explore comorbidities of BN with ADHD and BPD as well as the contribution of impulsivity as an underlying trait linking these disorders.

**Methods:**

Hundred and fifteen females with BN and 98 healthy matched controls (HC) (age range between 16 and 48 years) were assessed regarding adult and childhood-ADHD, personality disorders and impulsivity.

**Results:**

Patients with BN were more impulsive (*p* < 0.001) and more often fulfilled criteria of childhood/adulthood ADHD (*p* < 0.001) than HC, and criteria of BPD than expected in the general population. Childhood-ADHD (*p* = 0.009) and BPD (*p* = 0.017) both were significant positive predictors for impulsivity scores found in patients with BN.

**Conclusion:**

Comorbidity with ADHD and BPD often is prevalent in BN and associated with an increase in impulsivity, the latter being a relevant transdiagnostic trait. It might be beneficial to explore impulsivity as well as comorbidities in the clinical care of patients with BN.

**Level of evidence III:**

Evidence obtained from well-designed cohort or case–control analytic studies.

## Introduction

Bulimia nervosa (BN) is a serious mental illness with severe psychosocial as well as medical complications [1–3]. Apart from binge eating episodes and purging behaviour, a contributing factor to the aetiology, illness severity, maintenance and treatment success is impulsivity [4, 5]. Attention deficit hyperactivity disorder (ADHD) and borderline personality disorder (BPS) are common comorbidities of BN, all three illnesses sharing impulsivity as important part of their psychopathology.

Comorbid BN and ADHD have been investigated extensively over the past years [6, 7], and comorbidity rates are estimated at 31% [8] to 35–37% [9]. BN and ADHD show common aberrations regarding neurobiological and neuropsychological factors including impulsivity [10]. The symptom burden of comorbid patients seems to be greater for disordered eating as well as ADHD symptoms including impulsivity [8, 11]. Regarding BPS, 75% of patients with BN suffer from any personality disorder, mostly of BPS (25–47%) [7, 12, 13]. McDonald [14] reviewed the evidence about common genetic and epigenetic traits of BN and BPD and suggested that comorbid patients might even be a genetically and epigenetically different subgroup of patients.

The commonalities across different mental illnesses highlight impulsivity as trait of interest for transdiagnostic approaches [15], such as the RDoc framework [16]. Projects, such as the RDoC, strive towards a biologically based taxonomy for mental illnesses, rather than using symptom-based clusters [17]. Currently, impulsivity is included as a behavioral indicator for the subconstruct “cognitive control” linking it to impaired cognitive control, response selection and inhibition/suppression. Impulsivity is strongly influenced by genetics [18, 19] and has already been proposed as endophenotype in BN [10, 20].

Although it is well known that BN, ADHD and BPS share impulsivity as common trait, its clinical implications in the face of two or more of those mental illnesses is still uncertain. Impulsivity might be a distinct trait marker, having an additive effect or, alternatively, potentiate the symptom burden of patients with comorbid impulsivity related psychiatric illnesses. In this study, we wanted to further clarify the association between the presence of BN, ADHD, and BPS and the extent of impulsivity traits. Up to now, no study has examined the role of both ADHD and BPD, as relevant comorbidities of BN, and impulsivity traits in the same group of patients, although the relevance of this topic has already been established over a decade ago [21].

We assessed the prevalence of current and childhood-ADHD in a sample of patients with BN in comparison to healthy controls (HC). We furthermore compared impulsivity traits between patients with BN to HC. We also determined the prevalence of BPS in the group of BN patients, and, compared impulsivity traits between patients with and without childhood-ADHD, and with and without BPS. We divided the patient group regarding the reported presence or absence of childhood-ADHD, not current ADHD, to prevent confounding current ADHD symptomatology with the psychopathology of comorbid diseases such as BN or BPD [8], knowing that, even without fulfilling the criteria for ADHD as an adult, childhood-ADHD is a known risk factor for the development of other mental illnesses later in life [7, 22].

We expected that patients with BN have a high rate of retrospect childhood-ADHD [23, 24], and BPD [7, 22]. We hypothesized that patients with BN plus childhood-ADHD and/or BPS would have higher impulsivity rates than patients with BN alone, and that patients fulfilling all three diagnostic criteria would suffer from higher impulsivity scores than patients with only two of them.

## Materials and methods

### Study sample

Between 2008 and 2010, we recruited 115 females (age range = 16–47 years) with BN who were inpatients at the Schoen Clinic Roseneck in Prien, which offers specialized inpatient treatment for eating disorders. Inpatient treatment meant that outpatient treatment had not been possible or had not succeeded. Treatment included cognitive behavioural therapy, regular meals, as well as additional therapies, such as occupational therapy and physiotherapy. Exclusion criteria were intellectual disability, all diseases and injuries of the central nervous-system, psychosis, and acute suicidality. The exclusion criteria were chosen, because their presence could potentially add a confounding influence on attention performance and impulsivity or would mean that a patient was too ill to be burdened with participating in a study.

Additionally, we recruited a non-clinical control group with 98 healthy females (age range = 16–48 years). To ensure the absence of an eating disorder, the healthy controls completed the Eating Attitude Test (EAT) [25], and were excluded if they scored above a threshold of 20. Other mental pathologies were excluded by administering parts of the SCID-I [26] and the German version of the Patient Health Questionnaire [27].

BN and HC groups were comparable with regard to age and cognitive performance, measured as multiple-choice vocabulary (MCV) and the word fluency tests which we took as equivalents for an intelligence measure. See Table 1 for sample characteristics.

**Table 1:** Characteristics of study sample

	BN			HC			
	*n*	Mean (SD)		*n*	Mean (SD)		*p*
Age (years)	111	26 (7.19)		98	26 (7.4)		0.98
MCV-A	114	29.1 (3.5)		98	29.5 (3.1)		0.43
Fluency	114	38 (8.2)		98	37.1 (7.5)		0.41
*Childhood-ADHD*							
WURS-K (total score)	105	27.2 (11)		95	18.6 (6.6)		< 0.001***
*Adult ADHD*							
ADHS-SB (total score)	113	16.8 (9.5)		98	6.3 (4.6)		< 0.001***
ADHS-SB inattention	113	6.8 (15.3)		98	1.9 (10.5)		< 0.001***
ADHS-SB hyperactivity	113	3.6 (14.1)		98	0.8 (10.4)		< 0.001***
ADHS-SB impulsivity	113	1.0 (10.4)		98	0.5 (10.3)		< 0.001***
WRI (total score)	63	26.6 (11.4)		98	8.4 (4.8)		< 0.001***

		% cut-off (absolute number)		% cut-off (absolute number)	*p*
*Childhood-ADHD*					
WURS-K		29.5 (31)		8.4 (8)	< 0.001***
*Adult ADHD*					
ADHS-SB		18.3 (21)		1 (1)	< 0.001***
WRI		42.6 (49)		1 (1)	< 0.001***
*Childhood and adult ADHD* WRI + WURS-K		14.8 (17)		1 (1)	< 0.001***
ADHD-SB + WURS-K		11.3 (13)		0	< 0.001***

Age, multiple-choice vocabulary test—version A (MCV-A) and fluency: two-sided Student’s t-tests; WURS-K, ADHS-SB and WRI: Mann–Whitney U-test (non-normal distribution) and Chi-square tests. HC: healthy CONTROLS,

*BN* patients with bulimia nervosa, *SD* standard deviation. Significant *p*-values are indicated by *-***

### Ethics statement

This study was conducted in accordance with the Helsinki Declaration. All patients and HC consented in writing to participate, and the local ethics committee approved the study.

### ADHD

Participants were administered an interview and completed questionnaires regarding current and former symptoms of ADHD: the Wender–Reimherr Interview (WRI) [28] the ADHD self-rating scale (ADHS-SB) to account for current ADHD symptoms [29] and the Wender Utah Rating Scale (WURS-K) to assess childhood-ADHD [30]. Please see Table 1 for information of subsample sizes, since not all participants returned a complete set of questionnaires.

The WRI is a structured interview consisting of 28 questions and seven subscales (attention deficit, hyperactivity, temperament, affect lability, emotional overactivity, disorganization, impulsivity), rated on a 3-point Likert scale (from 0–2). The criteria for an ADHD-diagnosis are met if inattention and hyperactivity are above threshold (≤ 2) as well as at least two other subscales [28].

The ADHS-SB is a questionnaire consisting of 22 items, rated on a 4-point Likert scale (0–3) that represent the domains inattention, hyperactivity, and impulsivity. The inattentive or the hyperactive/impulsive subtype are present when at least six inattentive or hyperactive/impulsive items are rated with scores of two or higher, respectively. The combined type is present when both above criteria are fulfilled [29]. Score height represents the extent of the symptom burden in the respective domain [29].

The WURS-K is a questionnaire consisting of 21 items asking retrospectively about ADHD symptoms between the ages of 8 and 10 years. Items are rated from 0–3 and the cut-off is 30 for a diagnosis of childhood-ADHD [28].

### Personality disorders

The participants with BN completed the SCID-II interview according to DSM-IV which measures personality disorders [12, 13].

Impulsivity was measured by the Barratt Impulsiveness Scale (BIS-11) [31]. The BIS-11 is a questionnaire consisting of 30 items on a 5-point Likert scale). A total score as well scores for six first-order factors can be calculated: attention, motor, self-control, cognitive complexity, perseverance, and cognitive instability. Furthermore, the ADHS-SB impulsivity subscale (see above) was used as additional measure.

### Statistical analyses

We performed all statistical analyses using SPSS^®^ Statistics 26 (IBM Corp. Released 2019. IBM SPSS Statistics for Windows, Version 26.0. Armonk, NY: IBM Corp).

We compared patients and HCs on measures of age, MCV, and word fluency using the two-tailed Student’s t-test. All further analyses were done with the Mann–Whitney U-test to account for non-normal distributions. Alpha was adjusted for multiple testing using Benjamini–Hochberg correction.

We compared total scores on the WURS-K, ADHS-SB and WRI, and frequency counts of the subjects in the BN and HC group exceeding the cut-off of each questionnaire (WURS-K, WRI and ADHS-SB), respectively. We furthermore determined the presence of personality disorder in the BN group and compared it with average values found in the literature [12, 13].

Within the BN group, we distinguished between those with childhood-ADHD and those without (according to WURS-K scores above or under 30), like our previous paper [8]. We furthermore compared the complete BN group and both subgroups regarding the presence of personality disorders.

We also compared the BN and HC groups as well as BN with and without ADHD and BPD subgroups, respectively, regarding BIS-11 overall and subscores as well as ADHS-SB scores.

**Fig. 1:**
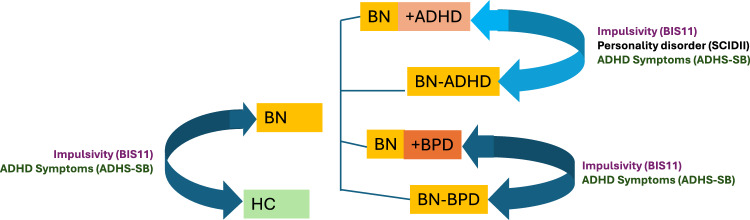
Overview of comparisons (depicted by arrows). Groups were compared regarding impulsivity, ADHD symptoms and presence of personality disorders. Subgroups are classified by presence or absence of comorbid ADHD or BPD: + = diagnosis present;− = diagnosis absent

Please see Fig. 1 for an overview of the comparisons.

Finally, we used multiple linear regression within the BN group with the dependent variable: BIS-11 total mean, and the predicting variables: presence versus absence of childhood-ADHD, presence versus absence of BPD, and age at interview. We wanted to further explore the predictors respective contribution to impulsivity.

## Results

### Childhood-ADHD and BPD in BN

Patients with BN fulfilled the criteria for childhood-ADHD significantly more often than HCs (OR = 4.6, 95%CI 1.98–10.52, p < 0.001), mainly due to inattention (see Table 2). Additionally, current ADHD symptoms were significantly more prevalent in patients with BN compared to HCs: BN participants fulfilled the criteria for current ADHD (ADHSSB: OR = 21.9, 95%CI: 2.89-166.24, *p* < 0.001; WRI: OR = 72.0, 95% CI 9.7–534.48, p < 0.001) as well as combined current ADHD plus childhood-ADHD (OR = 16.8, 95%CI: 2.2-128.92, *p* < 0.001) more often than HC. The width of the OR confidence intervals was probably due to the sample size. Any personality disorder was found in 24.3% of patients. Borderline personality disorder was the most prevalent with 11.7%, followed by Insecure personality disorder in 10.7% of patients with BN. None of the patients fulfilled criteria for Negativistic, Paranoid, Schizotypal, Schizoid, Histrionic, Narcissist, Antisocial, or other personality disorder.

**Table 2:** Personality disorders in the BN group

	BN	BN with childhood-ADHD	BN w/o childhood-ADHD	Subgroup
				Diff
Personality disorder	*n* = 115	*n* = 31	*n* = 74	corr. *p*
Insecure	12 (10.7%)	6 (19%)	4 (5.6%)	0.075
Dependent	1 (0.9%)	1 (3.2%)	0 (0.0%)	0.22
Obsessive–compulsive	4 (3.6%)	2 (6.5%)	2 (2.8%)	0.34
Depressive	7 (6.1%)	3 (9.7%)	3 (4.2%)	0.38
Borderline	13 (11.7%)	7 (23.3%)	5 (6.9%)	0.075

Rate of patients with BN meeting the SKID-II cut-off for a personality disorder and comparison of rate of personality disorders between patients with BN and childhood-ADHD versus BN without childhood-ADHD. Chi-square tests were administered

### Impulsivity

Patients with BN showed significantly higher impulsivity scores on the BIS11 (*p* < 0.001), most BIS11 impulsivity subscores (*p* = 0.001–0.119) and ADHS-SB impulsivity subscore (p < 0.001) but not self-control (*p* = 0.98), than HC (Table 3).

**Table 3:** Impulsivity in BN versus HC

	BN	HC	Diff
	Mean (SD)	Mean (SD)	corr. *p*
BIS 11	*N* = 98	*N* = 98	
Attention	11.3 (4.4)	7.9 (2.7)	< 0.001***
Motor impulsiveness	13.8 (4.5)	13.4 (3.7)	0.119
Self-control	11.8 (4.4)	11.1 (3.4)	0.98
Cognitive complexity	12.1 (3.8)	11.2 (2.9)	0.001***
Perseverance	6.8 (3.0)	6.3 (2.3)	0.029***
Cognitive instability	6.5 (2.8)	4.3 (1.8)	< 0.001***
Total score	63.3 (15.1)	54.7 (10.5)	< 0.001***

	N** = 113**	N** = 98**	
ADHS-SB impulsivity	1.0 (10.4)	0.5 (10.3)	< 0.001***

Mann–Whitney *U*-test (non-normal distribution) for mean impulsivity scores

FDR correction for multiple comparisons, significant corrected p-values are indicated by *-***

Patients with BN and childhood-ADHD exhibited significantly higher impulsivity scores on all BIS-11 domains except cognitive complexity (*p* < 0.001–0.038), as well as a higher ADHD symptom burden (*p* < 0.001) than patients without childhood-ADHD. Patients with comorbid BN and BPD scored significantly higher on all impulsivity scores (*p* < 0.002–0.035) other than self-control and cognitive complexity, and higher on ADHD total (*p* = 0.013), as well as all subscores (*p* = 0.003–0.015) other than inattention (Tables 4 and 5).

**Table 4:** Impulsivity in BN with versus without childhood-ADHD and BN with versus without BPD

	BN with childhood-ADHD *N* = 25	BN w/o childhood-ADHD *N* = 63	Subgroup diff	BN with BPD	BN w/o BPD	Subgroup Diff
				*N* = 13	*N* = 97	
	Mean (SD)	Mean (SD)	*p*	Mean (SD)	Mean (SD)	corr. *p*
*Bis 11* Attention	13.8 (3.0)	11.0 (3.3)	< 0.001***	14.2 (2.0)	10.9 (4.5)	0.012*
Motor impulsiveness	15.4 (2.8)	14.0 (3.0)	0.032**	13.8 (4.5)	13.5 (4.5)	0.002**
Self-control	13.3 (3.2)	11.7 (3.3)	0.038*	11.7 (4.5)	11.5 (4.5)	0.075
Cognitive complexity	12.8 (2.1)	12.3 (2.4)	0.36	12.0 (3.8)	12.0 (3.9)	0.85
Perseverance	8.0 (2.3)	6.8 (1.8)	0.014*	8.5 (2.3)	6.6 (3.1)	0.033*
Cognitive instability	7.7 (1.7)	6.5 (1.6)	0.006**	8 (2.1)	6.3 (2.9)	0.035*
Total score	71.0 (10.3)	62.2 (10.9)	0.001***	75.1 (10.8)	61.8 (15.0)	0.006**

Mann–Whitney *U*-test (non-normal distribution) for mean impulsivity scores

FDR correction for multiple comparisons, significant *p*-values are indicated by *-***

**Table 5:** ADHD symptoms in BN with versus without childhood-ADHD and BN with versus without BPD

	BN with childhood-ADHD *N* = 31	BN w/o childhood-ADHD *N* = 74	Subgroup diff	BN with BPD *N* = 13	BN w/o BPD *N* = 97	SUBGROUP DIFF
	Mean (SD)	Mean (SD)	*p*	Mean (SD)	Mean (SD)	corr. *p*
ADHS-SB total score	24.2 (9.8)	14.2 (8.2)	< 0.001***	23.0 (9.9)	13.5 (18.6)	0.013*
ADHS-SB inattention	12.9 (6.0)	7.1 (5.2)	< 0.001***	11.3 (6.7)	6.1 (16.2)	0.141
ADHS-SB hyperactivity	7.3 (3.3)	4.6 (2.8)	< 0.001***	7.7 (2.9)	2.8 (15.1)	0.003**
ADHS-SB impulsivity	4.0 (2.8)	2.4 (2.2)	0.004**	4.0 (2.4)	0.5 (14.6)	0.015*

Mann–Whitney *U*-test (non-normal distribution) for mean impulsivity scores

FDR correction for multiple comparisons, significant *p*-values are indicated by *-***

Multiple regression analyses revealed that BIS total scores were significantly predicted by the model, which explained 19.2% of the variance (F (3, 84) = 6.42, p < 0.001). The presence of childhood-ADHD was a significant predictor for higher BSI scores (Beta = 0,28, *p* = 0.009). The presence of BPD also was a positive predictor (Beta = 0,25, *p* = 0.017) for BSI total scores. Age at interview did not contribute significantly (Beta = 0.12, *p* = 0.25).

## Discussion

As expected, we found a higher prevalence of childhood-ADHD and current and former ADHD symptoms in the patient group in comparison to HC, in line with previous findings [8, 9].

We could also replicate former findings, that patients with BN and childhood-ADHD have higher symptom scores in all subdomains of impulsivity as well as ADHD symptoms compared to patients with BN alone: concordantly, in a sample of adult female BN patients, those with childhood-ADHD showed not only more severely disordered eating patterns, but also higher scores on measures of general psychopathology than those without childhood-ADHD [8]. The association between BN and ADHD-pathology seems to be prevalent also in ADHD-patient samples: in a study of adolescent girls and boys, childhood-ADHD was associated with more BN symptoms, and childhood impulsivity was the best predictor for BN symptoms in girls [32]. That said, the symptom burden of impulsivity and inattention is high in patients with BN already, as has been shown in neuropsychological tests as well as self-report questionnaires [8].

In our sample, patients with BN showed a prevalence of 11.7% BPD. Thus, the prevalence was higher than that in the general population, which was about 9% for any PD when using the DSM-IV in Germany [33]. Patients with BN and BPD were more impaired on most domains of impulsivity as well as in ADHD symptoms than patients with BN alone, mostly in line with Reas [34], who reported higher impulsivity-related traits to distinguish between patients with BN (alone) and BP and comorbid BPD.

We furthermore found higher impulsivity scores in participants with BN, childhood-ADHD and BPD. One explanation might be a stronger underlying predisposition for impulsivity increasing the risk to develop more than one mental illness. Or each comorbidity could increase impulsivity due to the burden of managing multiple diseases. Besides the role of genes, environmental effects such as traumatic experiences can additionally contribute to both bulimia nervosa (BN) and borderline personality disorder (BPD) [35, 36]. The clinical overlap of mental disorders with impulsivity as a core symptom, such as BN, ADHD, and BPD, has practical implications both for child and adolescent and adult psychiatrists. Due to the likelihood of a higher symptom burden of comorbid patients, it seems advisable to screen for severeness of impulsivity as well as for further impulsivity-related comorbidities in patients with BN.

Furthermore, with impulsivity as a core symptom of several mental illnesses, there might be a beneficial overlap of therapeutic approaches. The effectiveness of stimulant medication for the treatment of ADHD in childhood has long been established [37] and is the first line therapy in adults with ADHD [38]. The treatment of comorbid ADHD and BN with stimulants has shown to be effective, not only for the reduction of ADHD, but also for BN symptoms, albeit in rather small sample sizes [39–41]. Several studies hint at a direct effect of stimulant medication on bulimic and bingeing symptoms, even in the absence of ADHD. In case reports patients with BN without ADHD received psychostimulants successfully to reduce their bulimic symptoms [42]. A recent feasibility study revealed positive effects of lisdexamfetamine on binge episodes in patients with BN [43]. Furthermore, lisdexamfetamine was approved in the US in 2015 to treat mild to moderate binge eating disorder [44].

Comorbid BN and BPD suggests the implementation of dialectical-behavioural therapy (DBT), since it has proven to be effective in the treatment of both illnesses. It initially was developed for women with chronic suicidal ideation, mostly based on BPD [45], but which since has been adapted successfully for several other mental illnesses, amongst others BN [46]. DBT also proved effective in a sample of patients with comorbid BN and BPS [47].

### Strengths and limits

The strength of this study is the number of different diagnostic tools with which impulsivity and ADHD symptomatology were measured, and the fact that both, childhood-ADHD and BPS, were studied in combination with BN, whereas former studies usually have looked only at one of those two. Thus, we could explore these mental illnesses with the common trait impulsivity in more detail.

However, this study has some limitations. The sample size was too small to further investigate differences between the subgroups comprising combinations of childhood-ADHD, BN, and BPS. Furthermore, some data were only available for the BN group, preventing further comparisons with the HC group regarding certain personality traits, for example. The diagnosis of childhood-ADHD was given in retrospect, so that a certain memory bias cannot be fully excluded. To partially remediate this, we explicitly asked for childhood symptoms, to try and differentiate current, potentially overlapping symptoms from actual, longstanding diagnoses of ADHD. Finally, the data were collected in 2010, when the DSM-IV classification system was still valid, which impairs the possibility of comparing our results with studies using the DSM-5.

## Conclusions

In summary, impulsivity is a key symptom of BN, ADHD, and BPD. Comorbidity with at least childhood-ADHD and BPD often is prevalent in BN and associated with relevant additional impairments. It seems advisable to take these comorbidities into account when diagnosing and treating BN. It also might be helpful to target overlapping symptoms, such as impulsivity, early in the process. Finally, establishing symptom complexes rather than the existing diagnostic assignation to distinct illnesses, might be promising in better grasping the complexities of mental disorders.

### What is already known on this subject?

It is already known that AN, ADHD, and BPS share impulsivity as a common trait, and often occur comorbidly.

### What this study adds?

This study adds to a deeper understanding about the interplay of impulsivity and the presence of BN in combination with BPS and/or childhood-ADHD. Furthermore, it contributes to clinical considerations, such as screening patients with BN thoroughly for impulsivity-related comorbidities and taking these into account when choosing a therapeutic approach.

## Data Availability

No datasets were generated or analysed during the current study.

## References

[1] Nazar BP, Bernardes C, Peachey G et al (2016) The risk of eating disorders comorbid with attention-deficit/hyperactivity disorder: a systematic review and meta-analysis. Int J Eat Disord 49:1045–1057. 10.1002/eat.22643 27859581 10.1002/eat.22643

[2] Schmidt U, Adan R, Böhm I et al (2016) Eating disorders: the big issue. Lancet Psychiatry 3:313 27063378 10.1016/S2215-0366(16)00081-X

[3] Westmoreland P, Krantz MJ, Mehler PS (2016) Medical complications of anorexia nervosa and bulimia. Am J Med 129:30–37 26169883 10.1016/j.amjmed.2015.06.031

[4] Vaz-Leal FJ, Rodríguez-Santos L, García-Herráiz MA et al (2014) The role of depression and impulsivity in the psychopathology of bulimia nervosa. Revista de Psiquiatría y Salud Mental (English Edition). 10.1016/j.rpsmen.2013.06.002 10.1016/j.rpsm.2013.06.003 23972724

[5] Testa G, Granero R, Misiolek A et al (2022) Impact of impulsivity and therapy response in eating disorders from a neurophysiological, personality and cognitive perspective. Nutrients 14:5011. 10.3390/nu14235011 36501041 10.3390/nu14235011 PMC9738347

[6] Kaisari P, Dourish CT, Higgs S (2017) Attention deficit hyperactivity disorder (ADHD) and disordered eating behaviour: a systematic review and a framework for future research. Clin Psychol Rev 53:109–121. 10.1016/j.cpr.2017.03.002 28334570 10.1016/j.cpr.2017.03.002

[7] Leichsenring F, Fonagy P, Heim N et al (2024) Borderline personality disorder: a comprehensive review of diagnosis and clinical presentation, etiology, treatment, and current controversies. World Psychiatry 23:4 38214629 10.1002/wps.21156 PMC10786009

[8] Seitz J, Kahraman-Lanzerath B, Legenbauer T et al (2013) The role of impulsivity, inattention and comorbid ADHD in patients with Bulimia Nervosa. PLoS ONE 8:e63891. 10.1371/journal.pone.0063891 23700439 10.1371/journal.pone.0063891 PMC3659086

[9] Svedlund NE, Norring C, Ginsberg Y, von Hausswolff-Juhlin Y (2017) Symptoms of attention deficit hyperactivity disorder (ADHD) among adult eating disorder patients. BMC Psychiatry 17:1–9. 10.1186/s12888-016-1093-1 28095885 10.1186/s12888-016-1093-1 PMC5240294

[10] Levin RL, Rawana JS (2016) Attention-deficit/hyperactivity disorder and eating disorders across the lifespan: a systematic review of the literature. Clin Psychol Rev 50:22–36. 10.1016/j.cpr.2016.09.010 27693587 10.1016/j.cpr.2016.09.010

[11] Yao S, Kuja-Halkola R, Martin J et al (2019) Associations between attention-deficit/hyperactivity disorder and various eating disorders: a Swedish nationwide population study using multiple genetically informative approaches. Biol Psychiatry 86:577–586. 10.1016/j.biopsych.2019.04.036 31301758 10.1016/j.biopsych.2019.04.036 PMC6776821

[12] Braun DL, Sunday SR, Halmi KA (1994) Psychiatric comorbidity in patients with eating disorders. Psychol Med 24:859. 10.1017/S0033291700028956 7892354 10.1017/s0033291700028956

[13] Fahy TA, Russell IEGFM (1993) Personality disorder and treatment response in bulimia nervosa. Br J Psychiatry 162:765 8330109 10.1192/bjp.162.6.765

[14] Mcdonald S (2019) Understanding the genetics and epigenetics of bulimia nervosa/bulimia spectrum disorder and comorbid borderline personality disorder (BN/BSD–BPD): a systematic review. Eating Weight Disord Stud Anorexia Bulimia Obes 24:799–814. 10.1007/s40519-019-00688-7 10.1007/s40519-019-00688-7 PMC6751148 31119586

[15] Crisp ZC, Grant JE (2024) Impulsivity across psychiatric disorders in young adults. Compr Psychiatry 130:152449. 10.1016/j.comppsych.2023.152449 38184857 10.1016/j.comppsych.2023.152449

[16] Cuthbert BN (2020) The role of RDoC in future classification of mental disorders. Dialog Clin Neurosci 22:81–85. 10.31887/DCNS.2020.22.1/bcuthbert 10.31887/DCNS.2020.22.1/bcuthbert PMC7365298 32699508

[17] Cuthbert BN (2015) Research domain criteria: toward future psychiatric nosologies. Dial Clin Neurosci 17:89–97. 10.31887/dcns.2015.17.1/bcuthbert 10.31887/DCNS.2015.17.1/bcuthbert PMC4421905 25987867

[18] Hamilton PJ, Nestler EJ (2019) Epigenetics and addiction. Curr Opin Neurobiol 59:128 31255844 10.1016/j.conb.2019.05.005 PMC6889055

[19] Willems YE, Boesen N, Li J et al (2019) The heritability of self-control: A meta-analysis. Neurosci Biobehav Rev 100:324 30822436 10.1016/j.neubiorev.2019.02.012

[20] Howard M, Gregertsen EC, Hindocha C, Serpell L (2020) Impulsivity and compulsivity in anorexia and bulimia nervosa: a systematic review. Psychiatry Res 293:113354 32781364 10.1016/j.psychres.2020.113354

[21] Zapolski TCB, Settles RE, Cyders MA, Smith GT (2010) Borderline personality disorder, bulimia nervosa, antisocial personality disorder, ADHD, substance use: common threads, common treatment needs, and the nature of impulsivity. Indep Pract (Lutterworth) 30:20–23 21253443 PMC3022439

[22] Mishra S, Rawekar A, Sapkale B (2023) A comprehensive literature review of borderline personality disorder: unraveling complexity from diagnosis to treatment. Cureus. 10.7759/cureus.49293 38143629 10.7759/cureus.49293 PMC10748445

[23] Villa FM, Crippa A, Rosi E et al (2023) ADHD and eating disorders in childhood and adolescence: an updated minireview. J Affect Disord 321:265 36356347 10.1016/j.jad.2022.10.016

[24] Schiros A, Antshel KM (2023) The relationship between anorexia nervosa and bulimia nervosa, attention deficit/hyperactivity disorder, and suicidality in college students. Eur Eating Disord Rev 31:390. 10.1002/erv.2962 10.1002/erv.2962 36468533

[25] Garner DM, Garfinkel PE (1979) The eating attitudes test: an index of the symptoms of anorexia nervosa. Psychol Med 9:273. 10.1017/S0033291700030762 472072 10.1017/s0033291700030762

[26] Wittchen H-U, Zaudig M, Fydrich T (1997) Strukturiertes Klinisches Interview für DSM-IV (SKID I und SKID II)

[27] Saldivia S, Aslan J, Cova F et al (2019) Psychometric characteristics of the patient health questionnaire (PHQ-9). Rev Med Chil 147:53. 10.4067/S0034-98872019000100053 30848765 10.4067/S0034-98872019000100053

[28] Rösler M, Retz W, Retz-Junginger P et al (2008) Attention deficit hyperactivity disorder in adults. Benchmarking diagnosis using the Wender-Reimherr adult rating scale. Nervenarzt 79:320. 10.1007/s00115-007-2375-0 18210051 10.1007/s00115-007-2375-0

[29] Rösler M, Retz W, Thome J et al (2006) Psychopathological rating scales for diagnostic use in adults with attention-deficit/hyperactivity disorder (ADHD). Eur Arch Psychiatry Clin Neurosci 256:i3–i11. 10.1007/s00406-006-1001-7 16977549 10.1007/s00406-006-1001-7

[30] Retz-Junginger P, Retz W, Schneider M et al (2007) Der einfluss des geschlechts auf die selbstbeschreibung kindlicher ADHS-symptome. Nervenarzt 78:1046–1051. 10.1007/s00115-006-2242-4 17268790 10.1007/s00115-006-2242-4

[31] Vasconcelos AG, Malloy-Diniz L, Correa H (2012) Systematic review of psychometric proprieties of Barratt impulsiveness scale version 11 (BIS-11). Clin Neuropsychiatry 9

[32] Mikami AY, Hinshaw SP, Arnold LE et al (2010) Bulimia nervosa symptoms in the multimodal treatment study of children with ADHD. Int J Eating Disord 43:248. 10.1002/eat.20692 10.1002/eat.20692 19378318

[33] Maier W, Lichtermann D, Klingler T et al (1992) Prevalences of personality disorders (DSM-III-R) in the community. J Pers Disord 6:187. 10.1521/pedi.1992.6.3.187

[34] Reas DL, Pedersen G, Rø Ø (2016) Impulsivity-related traits distinguish women with co-occurring bulimia nervosa in a psychiatric sample. Int J Eating Disord 49:1093. 10.1002/eat.22606 10.1002/eat.22606 27567004

[35] Dodd DR, Crosby RD, Cao L et al (2022) Borderline personality disorder symptoms as mediational mechanisms linking childhood trauma and nonsuicidal self-injury among women with bulimia nervosa. Int J Eating Disord 55:372. 10.1002/eat.23669 10.1002/eat.23669 PMC8918001 34985154

[36] Utzinger LM, Haukebo JE, Simonich H et al (2016) A latent profile analysis of childhood trauma in women with bulimia nervosa: Associations with borderline personality disorder psychopathology. Int J Eating Disord 49:689. 10.1002/eat.22532 10.1002/eat.22532 PMC5268761 27038436

[37] Briars L, Todd T (2016) A review of pharmacological management of attention-deficit/hyperactivity disorder. J Pediatr Pharmacol Therapeut 21:192 10.5863/1551-6776-21.3.192 PMC4956327 27453697

[38] Young JL, Goodman DW (2016) Adult attention-deficit/hyperactivity disorder diagnosis, management, and treatment in the DSM-5 Era. Prim Care Companion J Clin Psychiatry 18:26599. 10.4088/PCC.16r02000 10.4088/PCC.16r02000 27907271

[39] Dukarm CP (2005) Bulimia nervosa and attention deficit hyperactivity disorder: a possible role for stimulant medication. J Womens Health 14:345. 10.1089/jwh.2005.14.345 10.1089/jwh.2005.14.345 15916509

[40] Ioannidis K, Serfontein J, Müller U (2014) Bulimia nervosa patient diagnosed with previously unsuspected ADHD in adulthood: clinical case report, literature review, and diagnostic challenges. Int J Eating Disord 47:431. 10.1002/eat.22231 10.1002/eat.22231 24311027

[41] Keshen A, Ivanova I (2013) Reduction of bulimia nervosa symptoms after psychostimulant initiation in patients with comorbid ADHD: five case reports. Eat Disord 21:360. 10.1080/10640266.2013.797828 23767675 10.1080/10640266.2013.797828

[42] Keshen A, Helson T (2017) Preliminary evidence for the off-label treatment of bulimia nervosa with psychostimulants: six case reports. J Clin Pharmacol 57:818. 10.1002/jcph.868 28111772 10.1002/jcph.868

[43] Keshen AR, Dixon L, Ali SI et al (2021) A feasibility study evaluating lisdexamfetamine dimesylate for the treatment of adults with bulimia nervosa. Int J Eat Disord. 10.1002/eat.23480 33534199 10.1002/eat.23480

[44] (2015) In brief: Lisdexamfetamine (vyvanse) for binge eating disorder. Medical Letter on Drugs and Therapeutics 57:25758546

[45] Hill DM, Craighead LW, Safer DL (2011) Appetite-focused dialectical behavior therapy for the treatment of binge eating with purging: A preliminary trial. Int J Eat Disord 44:249 20196109 10.1002/eat.20812

[46] Solmi M, Monaco F, Højlund M et al (2024) Outcomes in people with eating disorders: a transdiagnostic and disorder-specific systematic review, meta-analysis and multivariable meta-regression analysis. World Psychiatry 23:124–138. 10.1002/wps.21182 38214616 10.1002/wps.21182 PMC10785991

[47] Liakopoulou E, Vassalou G, Tzavara C, Gonidakis F (2023) A 12-month study of dialectical behavioral therapy for bοrderline patients suffering from eating disorders. Eating Weight Disord 28:81. 10.1007/s40519-023-01612-w 10.1007/s40519-023-01612-w PMC10556119 37798605

